# Influence of Atmospheric Cold Plasma Exposure on Naturally Present Fungal Spores and Physicochemical Characteristics of Sundried Tomatoes (*Solanum lycopersicum* L.)

**DOI:** 10.3390/foods11020210

**Published:** 2022-01-13

**Authors:** Junior Bernardo Molina-Hernandez, Jessica Laika, Yeimmy Peralta-Ruiz, Vinay Kumar Palivala, Silvia Tappi, Filippo Cappelli, Antonella Ricci, Lilia Neri, Clemencia Chaves-López

**Affiliations:** 1 Faculty of Bioscience and Technology for Food, Agriculture and Environment, University of Teramo, Via R. Balzarini 1, 64100 Teramo, Italy; jbmolinahernandez@unite.it (J.B.M.-H.); jlaika@unite.it (J.L.); yyperaltaruiz@unite.it (Y.P.-R.); vinaykumar.palivala@studenti.unite.it (V.K.P.); aricci@unite.it (A.R.); lneri@unite.it (L.N.); 2 Programa de Ingeniería Agroindustrial, Facultad de Ingeniería, Universidad del Atlántico, Carrera 30 Número 8-49, Puerto Colombia 081008, Colombia; 3 Department of Agricultural and Food Sciences, University of Bologna, 47521 Cesena, Italy; silvia.tappi2@unibo.it; 4 Inter-Departmental Centre for Agri-Food Industrial Research, University of Bologna, Via Quinto Bucci 336, 47521 Cesena, Italy; 5 Alma Plasma s.r.l., 40136 Bologna, Italy; filippo.capelli@almaplasma.com

**Keywords:** atmospheric cold plasma (ACP), sporicidal activity, *Aspergillus niger*, *Aspergillus rugulovalvus*, lycopene, antioxidant activity

## Abstract

This research aimed to evaluate the impact of atmospheric cold plasma (ACP) treatment on the fungal spores naturally present in sundried tomatoes, as well as their influence on the physico-chemical properties and antioxidant activity. ACP was performed with a Surface Dielectric Barrier Discharge (SDBD), applying 6 kV at 23 kHz and exposure times up to 30 min. The results showed a significant reduction of mesophilic aerobic bacteria population and of filamentous fungi after the longer ACP exposure. In particular, the effect of the treatment was assessed on *Aspergillus rugulovalvus* (as sensible strain) and *Aspergillus niger* (as resistant strain). The germination of the spores was observed to be reliant on the species, with nearly 88% and 32% of non-germinated spores for *A. rugulovalvus* and *A. niger,* respectively. Fluorescence probes revealed that ACP affects spore viability promoting strong damage to the wall and cellular membrane. For the first time, the sporicidal effect of ACP against *A. rugulovalvus* is reported. Physicochemical parameters of sundried tomatoes such as pH and water activity (*a_w_*) were not affected by the ACP treatment; on the contrary, the antioxidant activity was not affected while the lycopene content was significantly increased with the increase in ACP exposure time (*p* ≤ 0.05) probably due to increased extractability.

## 1. Introduction

The consumption of dried food products and the related risk to human health have increased globally, and concerns have been raised about the microbial quality of the products [1]. In fact, food security is considered a very important problem worldwide, and the potential effects of climate change on yields and quality of food crops, including mycotoxins, are of particular relevance. It is estimated that around 20–25% of harvested fruits and vegetables decompose during the post-harvest stage, even in developed countries [2]. In this context, tomato (*Lycopersicon esculentum* L. var. Excell and Aranca) is a very delicate and fragile vegetable, highly susceptible to microbial contamination, mechanical damage during transport, processing, and storage [3]. The most important dry-tomatoes producer in the world is China, followed by India, the United States, Turkey, Egypt, Iran, and Italy [4]. In 2018, 182 million tons were produced worldwide, of which Italy produced 4% of the total production [5]. Tomatoes are usually consumed fresh, but they are also processed into a diversity of foodstuffs, such as pulp, sauces, paste, juices, and dried tomatoes [6]. In particular, dried tomatoes production presents different advantages: It increases aroma, flavor, and shelf life, and reduces transportation costs thanks to the decrease in volume and weight [7]. However, drying is considered one of the critical points during the processing of dry tomatoes, because of the high microbial contamination found in the environment [6]. Contamination mainly comes from pathogenic or saprophytic fungi that are found both in soil and air and that have colonized food in the form of spores, probably carried by plant materials and packaging [8]. In this context, fungal spores are found ubiquitously and can tolerate many harsh conditions that are deleterious to many other life-forms such as austere temperatures, dryness, and high levels of radiation and radical exposure [9]. Thus, like other dry fruits, dry tomatoes could be susceptible to contamination with filamentous fungi such as *Aspergillus* spp., *Fusarium* spp., *Alternaria* spp., *Penicillium* spp., and their associated toxins during processing and storage [10]. The importance of fungal contamination in foodstuffs does not only refer to the possible degradative activity but also to the capability of many of them to produce mycotoxins to which humans are susceptible [11].

In recent years, novel food processing technologies have developed, each with peculiar strengths, with specific applications of the greatest significance for increasing foods shelf life, such as ohmic heating [12], high hydrostatic pressure, supercritical carbon dioxide [13], irradiation [14,15], and gaseous chlorine dioxide, alone and in combination with biological controls [16], ultraviolet treatment, and a pulsed electric field (PEF) [17]. Among these, cold plasma technology is gaining importance due to its effect in minimizing food-borne pathogens. Plasma can be generated under different conditions, both at low or at atmospheric pressure; the most exploited architectures use radiofrequency waves, microwaves, inductively coupled plasma (ICP), Dielectric Barrier Discharges (DBDs), and plasma jets. Among these, DBD and plasma jets are prominently used in food applications. A DBD consists of two parallel electrodes separated by a gap, and at least one of the electrodes must be covered by a dielectric layer. In this configuration, one of the electrodes is connected to a high-voltage generator while the other acts as the ground. As soon as the potential difference in the gap exceeds the breakdown voltage of the gas, the ionization process begins and micro-discharges are formed (plasma). However, to date, little research has addressed the cold plasma decontamination of dry fruit. In addition, since cold plasma contains various reactive species that can interact with food components, it is important to investigate the effects of such treatments on food quality [13,18,19]. Therefore, the aims of the present study were to (i) investigate the sporicidal effects of ACP treatments on the spores naturally present in sundried tomatoes and (ii) to evaluate the ACP impact on the physicochemical characteristics and antioxidant properties of the sundried tomatoes.

## 2. Materials and Methods

Sundried tomatoes (10 kg) were purchased from a marketplace at Teramo, Italy, in spring 2021, and stored in polyethylene bags at 4 °C until use.

### 2.1. Plasma Treatments

Atmospheric cold plasma (ACP) was generated by a Surface Dielectric Barrier Discharge (SDBD), where the setup included 4 rectangular high-voltage electrodes (115 cm^2^ each), and a mica dielectric layer over 2 mm thick. The ground electrode had the shape of a mesh, and it was in contact with the dielectric layer, whereas the plasma was formed in the holes of the mesh, producing an indirect treatment. The dry tomatoes were located under the ground electrode. A sinusoidal waveform was applied to the high-voltage electrode with a 6 kV of amplitude at 23 kHz.

ACP SDBD treatments were performed at room temperature (26 ± 1 °C). The sundried tomatoes samples were exposed to ACP treatment for 5, 10, 20 and 30 min and successively stored in polyethylene bags at 5 °C, until analysis. Each treatment was replicated three times in two different experiments. From each replicate, three sundried tomatoes samples were randomly selected (*n* = 9). In order to avoid introducing a new study variable (different batch contamination), all the experiment was repeated using the same sundried tomatoes batch. 

### 2.2. Changes in the Natural Microbiota Associate to Sundried Tomatoes after ACP

In order to determine the effect of ACP on the microbiota naturally associated with sundried tomatoes, treated and untreated samples were analyzed after treatment (t_0_) and after 22 days (t_22_) of storage (20 °C).

Random aliquots of 10 g sundried tomatoes, untreated and treated with cold plasma, were aseptically placed in sterile stomacher bags containing 90 mL of sterile physiological solution (0.9% *w*/*v*), and then successively homogenized in a Stomacher-400 Circulator (Seward, West Sussex, UK). Ten-fold dilutions from the homogenate were prepared, and 100 µL was inoculated on Plate Count Agar (PCA) for total mesophilic aerobic bacteria, Violet Red Bile Glucose Agar plates (VRBGA) for *Enterobacteriaceae* count, Violet Red Bile Agar plates (VRBA) for *coliforms* counts, Potato Dextrose Agar (PDA) for filamentous fungi and yeast, and DG18 for xerophilic filamentous fungi. Duplicate plates were made at each dilution. All the culture media were purchased from Liofilchem (Liofilchem, Roseto degli Abruzzi, Italy). PCA Plates were incubated at 30 °C for 48 h, PDA and DG18 agar plates for 5 days, and VRBGA and MRS plates at 37 °C for 48 h, with the last ones in anaerobic conditions. Coliforms were searched at 42 °C under microaerophilic conditions. The microbial counts were reported as logarithm colony-forming units per gram of sample (Log CFU/g). 

#### Phenotypical and Molecular Identification of Filamentous Fungi

A random number of each colony type was recovered from the different petri dishes of the untreated and ACP-treated sundried tomatoes. In order to identify the isolates on the basis of the morphological and growth characteristics, colonies were inoculated in two different culture media, malt extract agar (MEA) and Czapeck yeast extract agar (Liofilchem, Roseto degli Abruzzi, Italy). The isolation incidence of genus and species were calculated according to [20].

Subsequently, the molecular identification was determined according to the methodology reported by [21]. A PCR assay was performed using primers reported in Table 1. All genes were amplified using universal primers purchased from Sigma Aldrich (Saint Louis, MO, USA).

### 2.3. Preparation of Spore Suspensions 

In order to evaluate the resistance or sensitivity of the fungal spores to the ACP exposure, spores from *A. niger* and *A. rugulovalvus* were collected with sterilized physiological solution for five- (*A. niger*) and eight-day (*A. rugulovalvus* due to their low growth rate) cultures in MEA. Spores were optically standardized at a wavelength of 620 nm to obtain an optical density (OD) of 0.1 AU, corresponding to 3.0 × 10^4^ CFU/mL. The spore solution was diluted in a relation 1:2 with a sterilized physiological solution, and an aliquot (10 μL) was placed on sterile glass slides and dried with forced convection in a biosafety cabinet in sterility for 3 h. Afterward, glass slides were treated with cold ACP for 30 min. After treatment, 20 μL of malt extract broth (MEB) was added to the treated spore inoculation point successively, and the inoculated glasses slides were incubated in a humidity chamber for 24 h at 30 °C. Non-treated spores were considered the control. After that, spore germination was observed using a light microscope. Spores were considered germinated when their germ tube was longer than the same conidia [24]. All the experiments were realized in triplicate, and a total of 200 spores were counted for each sample.

#### Spore Viability after ACP Treatment 

In order to analyze the spore viability immediately after ACP treatment, treated and untreated spores from *A. rugulovalvus* (as sensible strain) and *A. niger* (as resistant strain) were stained with a mixture of CFDA (carboxyfluorescein diacetate) and propidium iodide (PI). While the green fluorescent dye CFDA is able to permeate both intact and damaged cell membranes, the red fluorescent dye propidium iodide (PI) can enter only the cells with significant membrane damage [25]. A 10^4^ spore solution in PBS was treated using 30 min of ACP. Subsequently, spores were stained as reported by [25]. A Nikon A1R confocal imaging system (Nikon Corp., Tokyo, Japan) was used to observe the spore viability.

### 2.4. Moisture, Water Activity and pH

Moisture content was measured according to the gravimetric method [26]. Water activity (*a_w_*) was determined using a hygrometer AquaLab CX 4-TE (Decagon Devices Inc., Pullman, WA, USA). The pH values were determined with a laboratory pH-meter (MP220, Mettler Toledo International, Polaris Parkway, OH, USA). All measurements were performed in triplicate.

### 2.5. Color Analysis 

The tomato color was evaluated in the epicarp area before and immediately after the ACP treatments. The analysis was conducted using a Konica Minolta Chroma Meter CR-5 spectrophotocolorimeter (Konica Minolta, Osaka, Japan) equipped with a D65 illuminant. Measurements were performed directly on a target mask with a measurement area of 8 mm using standard 10° observers. For each sample, 9 different halves were analyzed, and for each half, the colorimetric CIELab coordinates were collected in two different areas. L*, b*, and a* coordinates were thus used in order to calculate the redness (a*/b*), the hue angle (h°), and Chroma (C*), according to the following equations:

h° = arctan^−1^(b*/a*); (1)

C* = (a^2^ + b^2^)^0.5^; (2)

### 2.6. Lycopene Determination

Lycopene content was measured according to the methodology described by [27] with some modifications. Briefly, 0.5 g of sundried tomato were added to 5 mL of distilled water and homogenized for 5 min, using a dispersing homogenizer (Ultra-Turrax, Yellowline DI25basic, IKA, Staufen, Germany). After mixing, 10 mL of n-Hexane was added to the solution vortexed and centrifuged for 10 min at 4000 rpm (4 °C) (NEYA 16R centrifuge, Remi, Italy). The supernatant was recovered, and the extraction step was repeated until complete discoloration of the pellet, adding 5 mL of hexane. The absorbance of the lycopene dissolved in hexane was detected at 503 nm using a spectrophotometer (Lambda 25 UV/VIS, Perkin Elmer, MA, USA) and the results were expressed in μg lycopene/g of the sample, using the formula reported by [28]:

Lycopene(μg/g) = ((A_503_/ε × b) × MW × V/M) × 103 (3)

where:

A_503_ = absorbance of the hexane phase at 503 nm;

ε = molar extinction coefficient (L mol^−1^ cm^−1^) in the appropriate solvent;

b = cell optical path (cm);

MW = lycopene molecular weight = 536.9 g mol^−1^;

V = volume of hexane (mL);

M = sample mass (g).

### 2.7. Determination of the Antioxidant Capacity

Antioxidant capacity was evaluated on sundried tomatoes before and after ACP treatments using two different methods i.e., (i) the Folin–Ciocalteu (FC) assay, which is based on a redox reaction (single electron transfer—SET) and measures the capability of an extract to reduce a mixture of phosphomolybdic/phosphotungstic acid complexes (FC reagent) in alkaline medium, and (ii) the TEAC (Trolox Equivalent Antioxidant Capacity) assay that presents a mixed mechanism of action towards antioxidants since ABTS radicals can undergo reduction by both single electron transfer (SET) and hydrogen atom transfer (HAT).

Sample extraction was performed by adding an aliquot (2.0 ± 0.2 g) of sundried tomatoes to 10 mL of MeOH:H_2_O (80:20; *v*/*v*). Homogenization was performed for 5 min at 13,500 rpm using a dispersing homogenizer (Ultra-Turrax, Yellowline DI25basic, IKA, Staufen, Germany). The sample obtained was thus centrifuged at 4000 rpm for 20 min at 4 °C (NEYA 16R centrifuge, Remi, Italy) and the recovered supernatant was immediately used for analyses.

The FC assay was carried out using the method reported by [29] with modifications. In brief, 120 µL of extracts was added to 600 µL of diluted Folin–Ciocalteu reagent (1:10) and vortexed. After 2 min, 960 µL of Na_2_CO^3^ (7.5%) was added and incubated for 5 min in a water bath at 50 °C. The absorbance was measured with a spectrophotometer at 760 nm (Lambda 25 UV/VIS, Perkin Elmer, Waltham, MA, USA). The final values were calculated in equivalents of gallic acid, according to the calibration curve of the method. Analyses were performed in triplicate.

The TEAC assay was performed according to [30], with slight modifications. ABTS^•+^ (2,2′-azino-bis-(3-ethylbenzothiazoline-6-sulfonic-acid) was dissolved in water to a 7 mM concentration; the ABTS radical was formed by reacting ABTS stock solution with 2.45 mM potassium persulphate (K_2_S_2_O_8_) and allowing the mixture to stand in the dark at room temperature for 12–16 h. Before use, this solution was diluted with distilled water to obtain an absorbance of 0.700 ± 0.020 at 734 nm. The reaction was performed by mixing in cuvettes 30 µL of tomato extracts with 2970 µL of the ABTS^•+^ solution. The reaction mixture was incubated in the dark for 7 min at room temperature and the percentage of discoloration was used as the measure of antioxidant activity. The percent scavenging of ABTS^•+^ (cation radical) was calculated as:

(Absorbance Control − Absorbance sample)/Absorbance control × 100(4)

The antioxidant activity was expressed as μmol TEAC (Trolox Equivalent Antioxidant Capacity)/g tomato (dry matter). The TEAC value was calculated by the ratio of the regression coefficient of the dose–response curve of the sample and the regression coefficient of the dose–response curve of Trolox and was expressed as µmoles of Trolox equivalents per g of dry matter. The analysis was carried out on each extract in triplicate.

### 2.8. Statistical Analysis

Data were expressed as mean and standard deviation calculated on three replicated treatments and additionally analyzed by one-way ANOVA analysis. Significant differences between means were computed by Tukey’s multiple comparison test at a significance level of *p* ≤ 0.05. Data were processed using STATISTICA 12 for Windows (StatSoftTM, Tulsa, OK, USA) software.

## 3. Results 

### 3.1. Plasma Gas and Reactive Species Analysis

The literature regarding ACP is accurate about the chemistry that governs the atmosphere inside a plasma reactor, and depending on the surface power density (SPD) absorbed by the plasma, two different regimes may occur [31,32]. For small values of SPD, the chemistry is governed by the ozone formation reactions, and in this configuration, the concentration of ozone increases with time, eventually reaching a plateau. This regime is known as the ozone regime, and under these circumstances, the main reactive species that can be found in an ACP are ozone and atomic oxygen, both with a relevant antimicrobial effect [33,34].

Increasing the SPD leads to a greater formation of ozone up to a certain threshold, and once this value of SPD is exceeded, the NO_x_ formation reactions begin [34]. This regime is known as the transition regime. In fact, ozone has a transitory behavior: At first. the ozone concentration increases, reaches a maximum, and then decreases until it disappears. Many reactive species characterize this transition-regime: NO, N, NO_2_, NO_3_, N_2_O_5_, O, O_3_.

To address the regime of the plasma process under consideration, two long-living species were measured: Ozone and nitrogen dioxide. The ozone concentration increased during the whole treatment and no NO_2_ was detected; both results led to the conclusion that the performed process occurs in an ozone regime. 

The plasma source used in the present study presents the peculiarity that the plasma is confined in the holes of the mesh, therefore it is safe to assume that the greater part of ions and electrons are confined in these holes too. Furthermore, the short-living species (such as O and OH) do not have the time to reach the substrate to be treated due to their slow diffusion velocity. 

These considerations together with the fact that the plasma process occurs in the ozone regime led us to the conclusion that the main reactive species responsible for the biological effect is ozone.

The concentration of ozone was measured using the optical absorption spectroscopy technique that exploits the Lambert–Beer law. The behavior of O_3_ showed a strong increase, which reached a concentration of 350 ppm in 100 s; after this first phase, the concentration growth continued with a less steep trend, and the concentration reached after 600 s was about 600 ppm; at the end of the treatment, the concentration was about 900 ppm. The high-voltage generator (AlmaPlasma s.r.l., Bologna, Italy) was specifically designed for this application. 

### 3.2. Effect of Atmospheric Cold Plasma on the Total Mesophilic Aerobic Bacteria and Filamentous Fungi Count Naturally Present in Sundried Tomatoes

Natural Mesophilic Aerobic Bacteria (MAB) and filamentous fungi counts in sundried tomatoes at 5–30 min of ACP treatment periods compared to the untreated ones (control) are presented in Table 2. In the untreated samples, MAB and filamentous fungi were detected in low levels (3.06 ± 0.78 log CFU/g and 1.7 ± 0.82 log CFU/g, respectively) that were considered acceptable. Lactic acid bacteria, Coliforms, and *Enterobacteriaceae* were not detected in the analyzed samples. The absence of coliforms and *Enterobacteriaceae* could indicate the good hygienic and handling practices used to produce sundried tomatoes. 

The application of ACP for 10 min allowed us to obtain a slight (0.76 ± 0.32 log CFU/g) but significant reduction (*p* ≤ 0.05) of MAB, whereas further exposure at 20 and 30 min, did not show additional improvement. It is known that the conditions of availability of water activity found in a product such as sundried tomatoes do not allow the development of bacteria; however. foodborne pathogens and spores from bacteria and filamentous fungi have the potential to survive for an extended period on dried fruits [35]. In our study, we found that the *Bacillus* genus was the predominant bacterial population in the sundried tomato samples, as indicated by the presence of endospores in the isolated colonies grown in PCA that were evidenced by microscopically analysis. Studies on the efficacy of ACP against bacteria have been conducted overall in their vegetative states; however, there is little information on its potential for bacterial spore inactivation [36]. For example, a study conducted on *Bacillus cereus* and *Bacillus anthracis* spores revealed that 6 log CFU/g spores treated in liquid or air-dried on a solid surface were efficiently inactivated within 1 min of DBD plasma treatment at a discharge power of 0.3 W/cm^2^ [37]. On the other hand, other researchers obtained an inactivation of 3.5 to 4.8 log CFU/g of *B. subtilis* wild-type spores after 7 min of exposure to ACP; however, the inactivation depended on the process gas used [38]. In the present experiment, we used ambient air, which during the process generated high quantities of O_3_ (at 10 min is near 600 ppm), which were, however, not enough to kill spores. The small spore reduction observed in the present experiment might be explained by a different qualitative and quantitative composition of the plasma discharge and by the different sensitivities of the specific spore types [37]. Some authors suggested that the primary mechanism of spore inactivation is the diffusion of ROS (e.g., H_2_O_2_) into spores, followed by the damage of internal macromolecules or molecular systems. On the other hand, it has been reported that Alpha/beta-type small, acid-soluble proteins (SASPs) contribute to the resistance of spores to ACP and that the spores’ coat represents a protective layer against many oxidizing agents [38,39]. In addition, Patil et al. [40] reported that detoxifying enzymes present in the bacteria coat of the spore play an important role in detoxifying chemicals. 

With regard to the filamentous fungi counts, inactivation due to ACP was slightly higher compared to *Bacillus* spores. In fact, immediately after the 30 min treatment, a reduction of 1.35 log CFU/g was observed. As mentioned before, the comparison of the efficacy of the ACP treatment with previous studies is very difficult because it depends on the voltage, exposure time, initial microbial density, process gas, working distance, and plasma exposure [41]. However, in other studies performed on blueberries, cherries, tomatoes, and meat, the authors also reported a small reduction (from 0.8 to 1.5 log CFU/g) of yeast and filamentous fungi after ACP treatment [42,43,44]. In general, information on the sensitivity of fungal spores to plasma is scarce and focused on the decontamination of seeds, nuts, and powdered food [45,46,47]. 

As expected, 22 days after the ACP treatment (t_22_), there were no significant differences (*p* ≥ 0.05) between the control and the treated samples. In fact, as observed in Table 2, the water activity in the samples did not increase during storage, therefore the spores present on the sundried tomatoes surface were not able to germinate or form mycelia. 

### 3.3. Effect of the CAP on the Fungal Species

In order to verify if ACP treatments could have a species-dependent effect, the filamentous fungi associated with treated and untreated samples were identified. From 58 fungal isolates, a total of 23 different colonies of fungal morphotypes were found in the analyzed samples, but only 4 different genera were identified. In particular, *Aspergillus* genera represented the vast majority (78.2%) of the fungal colonies, followed by *Rhizopus* (13.04%), *Corynascus* (4.34%), and *Cladosporium* (4.34%). All the isolated specimens were identified at the molecular level and the gene sequences obtained were aligned with the accession numbers of the NCBI BLAST database and reported with their respective ones (Supplementary Table S1). 

In our study, the ITS gene was able to discriminate only four species, and for this reason we used other genes (*BenA, CaM*,) that gave us more information (Table S1). As evidenced, untreated sundried tomatoes harbored eight different species, such as *Aspergillus rugulovalvus* formerly *Aspergillus rugulosus* (syn *Emericella rugulosa* var. lazuline), *A. niger*, *A. amstelodami*, *A. tubingensis*, *A. cristatus*, *R. oryzae*, *Cladosporium cladosporioides*, and *Corynascus sepedonium*. As observed in Table S1, all *A. tubingensis* and *A. niger* strains, which belong to the *Nigri* section, were identified by means of amplification and sequencing of the *CamA* and *BenA* genes, respectively. These genes have been considered important for the identification of *Aspergillus* species, and in particular for the *Nigri* section [48]. It is important to underline that only *Aspergillus cristatus* was identified with the primers ITS1 and ITS4.

A great number of the species isolated here are of particular interest to food as they can grow rapidly over wide temperature and *a_w_* ranges (minimum ~0.70–0.72 *a_w_*). Moreover, the presence of *Aspergillus* ssp. is of special concern since several species of this genus are capable of producing mycotoxins, for example *A. rugulovalvus* potentially produce Sterigmatoxin; *A. niger* and *A. tubingensis* have the potential to secrete Ochratoxin A (OTA); and the xerophilic species *A. amstelodami* have been shown to produce multiple toxins, including Patulin, OTA, and Sterigmatocystin. In addition, other species identified here can produce mycotoxins during favorable conditions. For example, *R. oryzae* is a potential producer of Cyclopiazonic acid and Kojic acid, and *Cladosporium cladosporioides* has been reported to produce aflatoxins, Deoxynivalenol, Fumonisins, OTA, T-2 toxin, and Zearalenone. 

**Figure 1:**
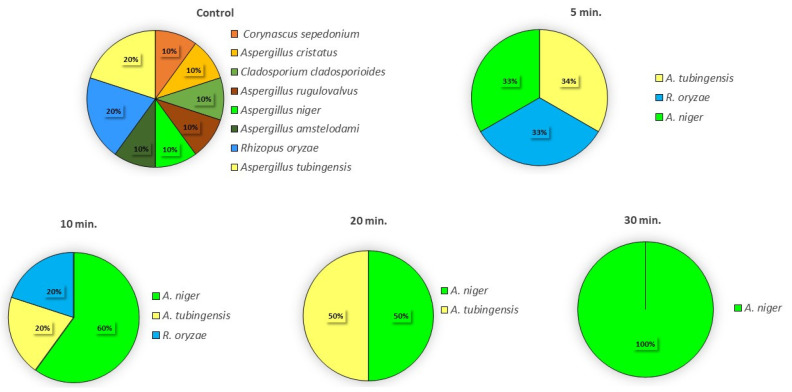
Incidence of the fungal species on sundried tomatoes before and after the ACP treatments.

As observed in Figure 1, ACP treatments affected the fungal community structure in sundried tomatoes, since fewer fungi were isolated, and their diversity was reduced with prolonged treatment of ACP. In fact, although significant differences were not found in colony counts after 5 min of ACP exposure with respect to the control samples, from eight species present in control samples, only three (*A. niger*, *A. tubingensis*, and *R. oryzae*) were detected in the treated samples with similar incidence. Similar behavior was observed even after 10 min of treatment, even if *A. niger* was isolated at a major frequency. After 20 min of treatment, only *A. niger* and *A. tubingensis* were isolated and in similar proportions. An interesting result was observed at 30 min of exposition. In fact, only *A. niger* colonies were isolated after ACP exposure. As stated in Section 2, it is important to underline that the greater length of the treatment implies a longer exposure of cells to O_3_. Thus, our results point out the efficacy of the treatment to inactivate spores of species that are frequently reported as a contaminant and able to produce important mycotoxins. Trompeter et al. [49] investigated the sporicidal activity of ACP using dielectric barrier discharges (DBDs) on *A. niger* spores, and reported that among different gases or mixtures of gases used (Ar, synthetic dry and moistened air, a mixture of O_2_ and O_3_ and N_2_, and N_2_ with 1% added H_2_), the argon discharge was proven to be the most efficient for spores’ inactivation. Recently, the efficacy of the high-voltage atmospheric cold plasma (HVACP) technology to reduce *A. flavus* spores was reported. In fact, about 50% spore inactivation was reached after 1 min of treatment [50].

**Figure 2:**
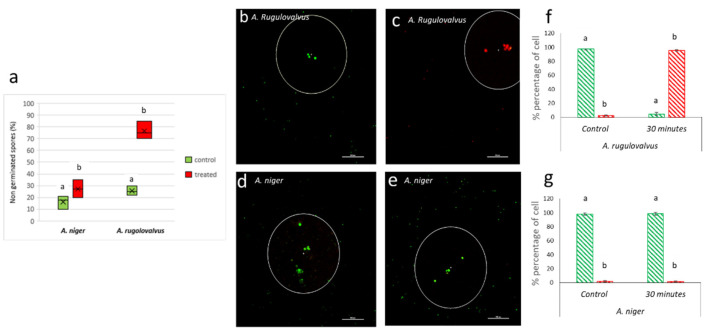
Effect of ACP on spore germination (**a**) of *Aspergillus niger* and *A. rugulovalvus* in malt extract medium after 30 min of ACP treatment. Confocal laser scanning microscopy analysis of cell viability in *A. rugulovalvus* (**b**,**c**) *A. niger* (**d**,**e**) after treatment and stained with green fluorescence CFDA (carboxyfuorescein diacetate) and red propidium iodide (PI) dyes. Bars indicate percentage of cell live (green) and death (red) spores in *A. rugulovalvus* (**f**) and *A. niger* (**g**), respectively. Different letters represent significant differences between the sample (*p* < 0.05; Tukey HSD post-hoc test).

In order to further demonstrate the effect of ACP on spore inactivation, spores of *A. niger* (the most resistant species) and *A. rugulovalvus* (most sensitive species) were subjected to the ACP treatment for 30 min. The spores were individually inoculated in vitro, and the germination percentage and vitality were measured 18 h after treatment. The results, presented in Figure 2a, show that the percentage of non-germinated spores was greater in *A. rugulovalvus* than in *A. niger,* confirming the higher resistance of *A. niger* spores to the stress induced by ACP. It is well known that multiple factors are able to inhibit fungal spores’ germination; in the present study, the disruption of the membrane permeability of the fungal spores was evaluated using confocal laser scanning microscopy coupled with CFDA and PI dyes. This methodology was already reported to be suitable for fungal spores analysis [51]. Our results showed that the inhibition of *A. rugulovalvus* spore germination was mainly due to the rupture of the spore permeability barrier after treatment. In fact, the use of nucleic acid stain labels such as propidium iodide (Figure 2b,c), which is unable to enter the nucleus in the intact spore, revealed a decrease in the amount of metabolically active spores after ACP. On the contrary, in treated spores of *A. niger* (Figure 2d), a small percentage was stained red, suggesting that this type of spore possess particular characteristics that allow their resistance against the oxidative stress generated by ozone and other ROS generated by ACP. In this context, some authors reported that the spore pigment melanin increases the firmness of spore cell walls, thus protecting the cell from stressors such as temperature and UV-irradiation, and increases the resistance to ROS, which are also produced in ACP [52]. Similarly, [53] suggested that melanization is a protection mechanism, due to the optical and antioxidant properties of melanin, which allow for limiting and repairing photodamage, due to electromagnetic energy or radiation that could remove electrons from water and other molecules (DNA and proteins) in the fungal cells. Other plausible hypotheses regarding the resistance of spores to ACP have been suggested by Montie et al. [54] who attributed it to their extremely thick polysaccharide cell walls that could represent an adaptation to environmental stresses [55]. On the other hand, as a result of the action of charged particles in ACP, the surface of the cell wall could be subjected to erosion that might cause damage to the cell wall structure and thickness [56].

### 3.4. Effects on Physicochemical Characteristics of Sundried Tomatoes

The physico-chemical characteristics of sundried tomatoes are presented in Table 3. As can be observed, after the ACP treatments, sundried tomatoes showed pH and *a_w_* values similar (*p* ≥ 0.05) to the control sample, while slight differences were observed in the moisture content, which decreased (loss < 6%) after 10 and 20 min of ACP treatment. This moisture loss can be attributed to the evaporation of superficial water due to convective flows triggered by the difference of temperature between the plasma source, which is about 30–35 °C, and the treatment chamber at room temperature (26 °C), and possibly promoted by cellular damages and surface modifications caused by plasma. In particular, ACP treatments were found to decrease the water contact angle of tomato peels and to accelerate the drying of tomatoes. Plasma-reactive species, in fact, can degrade the cuticle making its surface more hydrophilic, therefore increasing the water permeability of the plant surfaces and thus water diffusion from the plant interior [57]. Concerning the slight moisture increase highlighted after 30 min of treatment, it could be dependent on the accidental rehydration of the sample after ACP treatment rather than due to a higher initial moisture content of the sundried tomatoes sampled for this specific treatment.

As lycopene is one of the predominant carotenoids found in tomatoes [58], the effect of ACP on this compound was investigated. As shown in Table 3, ACP treatment positively affected the lycopene content leading to an increase of up to 42% after 20 min, while the extension of the treatment to 30 min resulted in a slight reduction. The increase in lycopene content upon ACP treatments was also observed by [58] and could be related to the enhancement of its extractability from the tomato skins as an effect of the cell damage induced by plasma-reactive species. On the other hand, the increase in the concentration of long-lifetime oxidizing species such as ozone in the treatment chamber could have been responsible for the lycopene reduction highlighted after 30 min of treatment. Overall, these results show that, after the previous optimization of the process parameters, cold plasma may be potentially used to enhance the extraction of lycopene from plant tissues.

As regards the antioxidant properties, no significant (*p* > 0.05) variation of the reducing power and of the radical scavenging activity was highlighted after the ACP treatments. Similar results were also reported by [59] on kiwifruit treated by cold plasma at 15 kV for 20 min; conversely, other studies showed a positive influence of ACP on the antioxidant properties of plant products, ascribing this effect to the formation of phenolic compounds with higher antioxidant capacity [60], to the increase in the extractability of polyphenols and other antioxidants such as carotenoids and vitamin C [61], and to the accumulation of secondary metabolites that include phenolic compounds as defense responses to UV irradiation [62,63]. It is important to consider that the instrument used in our study did not produce UV radiation. As highlighted by [64], different effects observed on the antioxidant properties of plant foods treated by cold plasma depend on the food matrix and on the plasma type, applied voltage, working gas, treatment time, and relative humidity. These factors, in fact, affect the types of reactive species generated, UV radiation, energetic ions, and charged particles that may induce physicochemical reactions with treated materials [65].

As color is probably the first quality factor judged by consumers on tomato products, the effect of ACP treatments on this attribute was also evaluated. As shown in Table 4, ACP did not affect the a* and b* values of tomato peels as well as the h°, C*, and a*/b* values, while we determined, irrespectively of the treatment time, a slight reduction (about 11%) of lightness (L*). This darkening could be due to possible melting of cutaneous wax on the peel surface [66].

## 4. Conclusions

The results obtained in the present study highlighted that the application of ACP has potential for the fungal spore decontamination of sundried tomatoes. As demonstrated, it exhibited several levels of the sporicidal effect depending on the types of spores and fungal species. In addition, this study demonstrated that the inactivation processes depend on the exposure time, which is related to the increase in the ozone in the ACP chamber, which reached about 900 ppm at the end of the treatment (30 min). In addition, fluorescence probes revealed that ACP affects spore viability considerably in *A. rugulovalvus,* which was associated with strong damage of the wall and cellular membrane of the spores. For the first time, the sporicidal effect of ACP against *A. rugulovalvus*, a species that produces the antifungal lipopeptide caspofungin B, has been reported. Concerning the physicochemical properties, ACP slightly affected the water content and the color of sundried tomatoes, but did not influence the antioxidant properties, while it increased the extractability of lycopene. In conclusion, the obtained results showed good potentiality of this technology for dry product decontamination and highlighted the importance of process optimization considering the effect on quality and nutritional properties.

## Figures and Tables

**Table 1:** Primers used for PCR assay.

Gene Target	Length bp	Primer	Sequences (5′ → 3′)	Reference
*Internal transcribed Spacer* (*ITS*)	420–825	ITS1 (F)	5′TCCGTAGGTGAACCTGCGG3′	[22]
		ITS4 (R)	5′TCCTCCGCTTATTGATATGC3′	
β-tubulin (*BenA*)	1125	β-tub 2a (F)	5′GGTAACCAAATCGGTGCTTTC3′	[22]
		β-tub 2b (R)	5′ACCCTCAGTGTAGTGACCCTTGGC3′	[23]
Calmodulin (*CaM*)	543	Cmd5 (F)	5′-CCGAGTACAAGGAGGCCTTC-3′	[23]
		Cmd6 (R)	5′-CCGATAGAGGTCATAACGTGG-3′	

Abbreviation: F: Forward, R: Reverse. BenA means B-tubulin and CaM mean calmodulin.

**Table 2:** Total mesophilic aerobic bacteria and filamentous fungi count naturally present in sundried tomatoes immediately (t_0_) and at 22 (t_22_) days after ACP treatment.

Time of Treatment (min.)	Mesophilic Bacteria (log UFC/g)		Filamentous Fungi (log CFU/g)	
	t_0_	t_22_	t_0_	t_22_
Control	3.06 ± 0.78 ^a^	3.12 ±0.54 ^a^	1.79 ± 0.82 ^a^	1.56 ± 0.75 ^a^
5	2.95 ± 0.45 ^a^	3.06 ±0.34 ^a^	1.68 ± 0.4 ^a^	1.54 ± 0.56 ^a^
10	2.30 ± 0.50 ^b^	2.40 ±0.10 ^b^	0.77 ± 0.45 ^a^	0.47 ± 0.52 ^a^
20	2.15 ± 0.28 ^b^	2.30 ±0.19 ^b^	0.62 ± 0.6 ^a^	0.67 ± 0.58 ^a^
30	2.20 ± 0.56 ^b^	2.10 ±0.64 ^b^	0.44 ± 0.52 ^b^	0.51 ± 0.61 ^a^

Mean and standard deviation of three repetitions in two different experiments. Different letters in the same line mean significant differences between the treatments (*p* < 0.05; Tukey HSD post-hoc test).

**Table 3:** Effect of ACP treatment on the physicochemical and antioxidant characteristics of sundried tomatoes. Treatments were carried out at ambient temperatures of 26 ± 1 °C.

Time of Treatment (min.)	pH	Moisture Content %	*a_w_*	Lycopene (g/g dw)	FC (mg GAE/g dw)	TEAC (µmol TE/g dw)
0	4.36 ± 0.059 ^a^	34.25 ± 1.57 ^a^	0.608 ± 0.006 ^a^	3.22 ± 0.05 ^d^	3.35 ± 0.16 ^a^	8.35 ± 0.55 ^ab^
5	4.34 ± 0.053 ^a^	33.29 ± 1.37 ^a^	0.610 ± 0.005 ^a^	3.58 ± 0.21 ^c^	3.58 ± 0.24 ^a^	7.58 ± 0.42 ^b^
10	4.30 ± 0.075 ^a^	29.00 ± 0.28 ^b^	0.610 ± 0.004 ^a^	4.13 ± 0.26 ^b^	3.57 ± 0.24 ^a^	9.28 ± 0.66 ^a^
20	4.25 ± 0.079 ^a^	29.86 ± 0.29 ^b^	0.611 ± 0.004 ^a^	4.57 ± 0.03 ^a^	3.35 ± 0.05 ^a^	7.51 ± 0.91 ^b^
30	4.29 ± 0.076 ^a^	32.43 ± 0.36 ^a^	0.613 ± 0.007 ^a^	4.14 ± 0.11 ^b^	3.48 ± 0.13 ^a^	7.89 ± 0.71 ^ab^

Mean and standard deviation of three repetitions in two different experiments. Different letters in the same line mean significant differences between the treatments (*p* < 0.05; Tukey HSD post-hoc test).

**Table 4:** Instrumental color in the CIE L*a*b* space.

Time of Treatment (min)	L*	a*	b*	C*	h°
0	29.20± 3.64 ^a^	19.74 ± 3.75 ^a^	15.20 ± 3.29 ^a^	24.94 ± 4.81 ^a^	37.48 ^a^ ± 3.14 ^a^
5	26.03 ± 5.79 ^b^	16.98 ± 4.76 ^a^	14.49 ± 5.25 ^a^	22.37 ± 6.92 ^a^	39.54 ^a^ ±4.47 ^a^
10	25.10 ± 3.27 ^b^	18.23 ± 4.68 ^a^	14.38 ± 3.94 ^a^	23.27 ± 5.92 ^a^	38.16 ^a^ ± 3.79 ^a^
20	24.07 ± 4.52 ^b^	17.45 ± 2.94 ^a^	12.91 ± 2.69 ^a^	21.71 ^a^ ± 3.93 ^a^	36.35 ^a^ ± 1.83 ^a^
30	25.92 ± 4.76 ^b^	18.63 ± 2.46 ^a^	14.72 ± 3.27 ^a^	23.78 ^a^ ± 3.81 ^a^	38.01 ^a^ ± 3.42 ^a^

L*: Luminosity index; a*: Green–red color coordinate; b*: Blue–yellow color coordinate; C*: Croma; h°: Hue. Data on the same column marked with different letters are significantly different at a *p* < 0.05 level.

## Data Availability

Not applicable.

## Supplementary Materials

The following supporting information can be downloaded at: https://www.mdpi.com/article/10.3390/foods11020210/s1, Table S1. Identification of the fungi isolates from dry tomatoes, determined by sequences of the ITS, β-tubulin, and Calmodulin genes.
