# Supplementary material for: Influence of Atmospheric Cold Plasma Exposure on Naturally Present Fungal Spores and Physicochemical Characteristics of Sundried Tomatoes (Solanum lycopersicum L.)
Source: Foods. 2022 Jan 13;11(2):210. doi: 10.3390/foods11020210 (PMC8774998; doi:10.3390/foods11020210)
Supplement: Supplementary file 1 [file foods-11-00210-s001.zip › foods-1515545-supplementary.pdf]

Supplementary Materials

Table S1. Identification of the fungi isolates from sun-dried tomatoes, determined by sequences of the ITS, β-tubulin, and Calmodulin gene.

Sample	Closest relative	Accession number	ITS1-ITS4	βt2a-βt2b	CMD5-CMD6
**VJT-1**	*Aspergillus niger*	**MT597823.1**	N.A.	99.12	N.A.
**VJT-6**	*Aspergillus niger*	**KY990205.1**	N.A.	98.8	N.A.
**VJT-17**	*Aspergillus niger*	**KU865178.1**	N.A.	99.7	N.A.
**VJT-14**	*Aspergillus niger*	**MN907662.1**	N.A.	99.7	N.A.
**VJT-15**	*Aspergillus niger*	**JX545078.1**	N.A.	99.7	N.A.
**VJT-19**	*Aspergillus niger*	**KJ36066.1**	N.A.	99.1	N.A.
**VJT-26**	*Aspergillus niger*	**MH447369.1**	N.A.	99.1	N.A.
**VJT-7**	*Aspergillus niger*	**HQ632731.1**	N.A.	N.A.	99.6
**VJT-12**	*Aspergillus niger*	**MN493772.1**	N.A.	99.1.	N.A.
**VJT-28**	*Aspergillus niger*	**LC577101.1**	N.A.	99.1.	N.A.
**VJT-18**	*Aspergillus niger*	**MTI23512.1**	N.A.	99.1.	N.A.
**VJT-8**	*Aspergillus tubingensis*	**KY612372.1**	N.A.	N.A.	98.9
**VJT-10**	*Aspergillus tubingensis*	**KY612372.1**	N.A.	N.A.	98.9
**VJT-16**	*Aspergillus tubingensis*	**MK166185.1**	N.A.	N.A.	98.9
**VJT-30**	*Aspergillus tubingensis*	**KX231824.1**	N.A.	N.A.	98.9
**VJT-2**	*Aspergillus cristatus*	**KY828916.2**	99.32	N.A.	N.A.
**VJT-5**	*Aspergillus rugulovalvus*	**AB248319.1**	N.A.	100	N.A.
**VJT-20**	*Aspergillus amstelodami*	**FR775356.2**	N.A.	99.7	N.A.
**VJT-3**	*Cladosporium cladosporioides*	**KY039309.1**	99.3	N.A.	N.A.
**VJT-4**	*Corynascus sepedonium*	**MK919294.1**	99.43	N.A.	N.A.
**VJT-11**	*Rhizopus oryzae*	**LC514321.1**	99.6	N.A.	N.A.
**VJT-22**	*Rhizopus oryzae*	**LC514326.1**	99.7	N.A.	N.A.
**VJT-25**	*Rhizopus oryzae*	**MT603963.1**	99.7	N.A.	N.A.

N.A.: Not amplified.
